# A Metabolic Shift toward Pentose Phosphate Pathway Is Necessary for Amyloid Fibril- and Phorbol 12-Myristate 13-Acetate-induced Neutrophil Extracellular Trap (NET) Formation*

**DOI:** 10.1074/jbc.M115.640094

**Published:** 2015-07-21

**Authors:** Estefania P. Azevedo, Natalia C. Rochael, Anderson B. Guimarães-Costa, Thiago S. de Souza-Vieira, Juliana Ganilho, Elvira M. Saraiva, Fernando L. Palhano, Debora Foguel

**Affiliations:** From the ‡ Instituto de Bioquímica Médica Leopoldo de Meis, Programa de Biologia Estrutural, Universidade Federal do Rio de Janeiro, Rio de Janeiro 21941-902 and; § Instituto de Microbiologia Paulo de Góes, Universidade Federal do Rio de Janeiro, Rio de Janeiro 21941-970, Brazil

**Keywords:** amyloid, glucose-6-phosphate dehydrogenase (G6PD or G6PDH), NADPH oxidase, neutrophil, pentose phosphate pathway (PPP)

## Abstract

Neutrophils are the main defense cells of the innate immune system. Upon stimulation, neutrophils release their chromosomal DNA to trap and kill microorganisms and inhibit their dissemination. These chromatin traps are termed neutrophil extracellular traps (NETs) and are decorated with granular and cytoplasm proteins. NET release can be induced by several microorganism membrane components, phorbol 12-myristate 13-acetate as well as by amyloid fibrils, insoluble proteinaceous molecules associated with more than 40 different pathologies among other stimuli. The intracellular signaling involved in NET formation is complex and remains unclear for most tested stimuli. Herein we demonstrate that a metabolic shift toward the pentose phosphate pathway (PPP) is necessary for NET release because glucose-6-phosphate dehydrogenase (G6PD), an important enzyme from PPP, fuels NADPH oxidase with NADPH to produce superoxide and thus induce NETs. In addition, we observed that mitochondrial reactive oxygen species, which are NADPH-independent, are not effective in producing NETs. These data shed new light on how the PPP and glucose metabolism contributes to NET formation.

## Teaser

**Background:** Neutrophil extracellular traps (NETs) are DNA meshes that snare and kill microorganisms, impeding their dissemination.

**Results:** Glucose but not fructose supports NET formation through a metabolic shift toward pentose phosphate pathway (PPP).

**Conclusion:** PPP impairment leads to decreased NET production.

**Significance:** This study provides novel knowledge about the mechanisms of NET induction, opening new avenues of study and intervention.

## Introduction

Neutrophils are highly specialized cells of the innate immune system that are the first to reach a site of infection (1). Neutrophils possess a myriad of microbicide mechanisms to deal with infection (1). Among them, neutrophils can release granules containing proteases and microbicide peptides, and produce extracellular and intracellular reactive oxygen and nitrogen species (ROS/RNS).^5^ These cells can also phagocytose microorganisms or release chromatin traps decorated with cytoplasm and granular proteins (1, 2) such as elastase, myeloperoxidase, etc. These chromatin traps are termed neutrophil extracellular traps (NETs), first described in 2004 by Brinkmann *et al.* (3) using bacteria as stimuli. Today, there is a vast range of data suggesting that not only bacteria, but viruses, fungi, protozoa, and non-microbial molecules are able to induce NET release from human neutrophils (2, 4). Our group showed for the first time (5) that *in vitro* amyloid fibrils (AF), which are insoluble, β-sheet-rich proteinaceous molecules (6), are potent NET inducers. Often, AF are associated with diseases, and their accumulation in different tissues causes inflammation and tissue degeneration, as described for the most common amyloid pathologies, Alzheimer and Parkinson diseases (6).

Our data showed that, once tangled within the NETs, AF are digested by NET-associated elastase forming small oligomeric species that are toxic to cells in culture. This suggests that AF may be a reservoir of toxic species in amyloidogenic diseases, and the NET-associated proteases might trigger their formation. We also observed the presence of NET co-localizing with AF in postmortem tissues of amyloidotic patients, suggesting that AF-induced NET formation can take place *in vivo* (5). However, there are no data regarding how fibrils induce NET formation.

Although the factors that regulate NET formation remain unclear for most NET-inducing agents, many groups have described the participation of myeloperoxidase (MPO (7, 8)), NO (9), and ROS such as superoxide in NET release (4, 10, 11). The production of ROS, which is necessary for NET formation, arises mainly from the cytosolic NADPH oxidase (Nox) (11, 12), a multicomponent membrane-associated enzyme. Nox can be directly activated by several protein kinases such as PKC through phosphorylation of the gp91^phox^ component (13). How the ROS signal induces NET formation is still unknown. Nox produces superoxide from oxygen using reduced NADPH as a cofactor (14). NADPH is mainly produced from NADP^+^-dependent dehydrogenases, such as glucose-6-phosphate dehydrogenase (G6PD), an important enzyme responsible for the first reaction of the pentose phosphate pathway (PPP) (15).

The importance of the PPP for neutrophil function is clearly observed in patients with G6PD deficiency or impairment, in which the development of infections is common due to dysfunctional neutrophil microbicide mechanisms (16, 17). The poor neutrophil function caused by this impairment is directly associated with less NADPH and with less ROS production (16). Some mutations in G6PD can severely affect the G6PD activity, causing the neutrophils and monocytes to be unresponsive to phorbol 12-myristate 13-acetate (PMA) and thus producing less ROS than wild-type cells (18). Although there are data showing that G6PD activity is important for ROS production (16–18), it remains unclear whether the PPP and specifically the G6PD activity are essential for NET production.

Herein we investigate whether human neutrophils stimulated by either AF or PMA depend on the PPP to generate the majority of the cellular ROS and induce NET formation. We demonstrate that mitochondrial ROS elicited by PMA or antimycin A/rotenone treatment are not effective in producing NETs from human neutrophils. To highlight the importance of PPP in NET formation, we demonstrate that G6PD activity increases after PMA or AF stimulation and when G6PD activity is blocked by the NADPH competitive inhibitor 6-aminonicotinamide (6-AN), NET formation is abrogated, reinforcing the role of PPP and glucose to neutrophil function in innate response.

## Experimental Procedures

### 

#### 

##### Neutrophil Isolation

The procedures using human biological samples were performed in accordance with institution regulations and approved by the Institutional Review Board for Human Subjects (CAAE#03102012.4.1001.5257; Hospital Universitário Clementino Fraga Filho, Universidade Federal do Rio de Janeiro, Rio de Janeiro, Brazil). Briefly, human neutrophils were isolated from buffy coats of healthy blood donors using a density gradient centrifugation (Histopaque, Sigma) as described (19).

##### Extruded NET-DNA Quantification

NET-DNA was quantified in the supernatant of 1 × 10^6^ human neutrophils using PicoGreen reagent (Invitrogen) as described (19). 2-Deoxyglucose (2-DG) at 2 mm was incubated with neutrophils for 3 h in RPMI 1640, and NET release was measured accordingly.

##### Amyloid Fibril Preparation

A25T, a highly amyloidogenic variant of transthyretin, was expressed heterologously and purified, and amyloid fibrils were grown as described (20). To ensure that no endotoxin was present, all recombinant proteins were further purified using a polymyxin-B-conjugated resin (Thermo Scientific).

##### Thioflavin-T Binding Assay and Transmission Electron Microscopy

Amyloid fibrils were diluted to 2 μm and incubated with 40 μm thioflavin-T (Sigma) for 5 min. Thioflavin-T fluorescence was read in a spectrofluorometer at 440 nm excitation and 480 nm emission and plotted using the GraphPad Prism 5.0 software (GraphPad Software). Amyloid was also characterized using transmission electron microscopy as described (5).

##### Immunocytochemistry

Human neutrophils were treated accordingly and then fixed with 4% paraformaldehyde for 20 min at room temperature. Cells were washed, and unspecific sites were blocked with 5% BSA for 1 h. Afterward, cells were incubated with primary antibodies such as anti-human MPO (1:150; Calbiochem) and anti-human elastase (1:150; Calbiochem) for 24 h at 4 °C. Cells were washed and incubated with secondary antibody donkey anti-mouse Alexa Fluor 488 (1:500, Invitrogen) and goat anti-rabbit Alexa Fluor 555 (1:500; Invitrogen) and mounted using Prolong antifade reagent (Invitrogen). Images were obtained using a confocal microscope Leica TCS SPE (Leica, Germany) and processed using the LAS AF Lite and Adobe Photoshop 7.0 software.

##### Flow Cytometry

Freshly isolated human neutrophils were incubated in RPMI 1640 without phenol red with Akt IV inhibitor (Akti; Calbiochem) at 10 μm, 6-AN at 10 mm, rotenone (Rot) at 2 μg/ml, diphenyleneiodonium (DPI) at 10 μg/ml, and antimycin A (AA) at 1 μm for 30 min at 37 °C, 5% CO_2_. All inhibitors were purchased from Sigma, unless stated otherwise. For experiments testing different fuel sources, we incubated neutrophils in Hanks' balanced salt solution (HBSS) (0.137 m NaCl, 5.4 mm KCl, 0.25 mm Na_2_HPO_4_, 0.44 mm KH_2_PO_4_, 1.3 mm CaCl_2_, 1.0 mm MgSO_4_, and 4.2 mm NaHCO_3_, pH 7.3, and filtered using 0.22-μm -filters). HBSS was supplemented with d-glucose (5.5 mm) or d-fructose (5.6 mm) or used without supplementation (no carbon source (NCS)). Afterward, dihydrorhodamine-123 (DHR, 1.2 μm, Sigma), annexin V, and MitoSOX were added, and then neutrophils were stimulated with PMA at 100 nm or A25T-AF at 3 μm for 15 min at 37 °C, 5% CO_2_. The fluorescence intensity of individual cells was analyzed by flow cytometry with a FACSCalibur flow cytometer (BD Biosciences). Graphs were plotted as mean fluorescence intensity (MFI) or the percentage of cells that were DHR^+^ (percentage of DHR-positive neutrophils) and MitoSOX-positive neutrophils.

##### Measurement of G6PD Activity

G6PD activity was measured according to Ref. 21. 2 × 10^7^ neutrophils were lysed at −80 °C and vortexed, and cell debris was separated by centrifugation. Protein content was measured using the DC protein assay (Bio-Rad). Briefly, 0.055 m Tris-HCl, 0.0033 m MgCl_2_, pH 7.8, 0.006 m NADP^+^, 0.1 m glucose 6-phosphate were added to each well in a clear bottom 96-well plate and incubated at 30 °C for 10 min to equilibrate and to establish a blank rate. Then, 3.3 μl of neutrophil lysates were added to wells, and optical density at 340 nm was monitored using a plate reader for 30 min at a 1-min rate. As controls, a well containing no lysate and a well containing purified recombinant G6PD (Sigma) were also monitored during experimentation. Enzyme activity was measured according to the following equation: units/mg = (Δ*A*_340 nm/min_)/6.22 × mg of protein/ml of reaction volume.

##### Intracellular ATP Quantification Assay

Intracellular ATP was measured in neutrophil lysates using the Mitochondrial ToxGlo kit (Promega) according to the manufacturer's instructions.

##### Intracellular Pyruvate Quantification Assay

Intracellular pyruvate production was assessed using a commercial pyruvate measuring kit according to manufacturer's instructions (Abcam).

##### Lactate Dehydrogenase (LDH) Viability Assay

Human neutrophils were incubated for 5 h in HBSS alone or supplemented with 5.5 mm glucose or 5.6 mm fructose, in RPMI 1640 with 10 mm 6-AN or with 1% Triton X-100 as a positive control, and 50 μl of neutrophil supernatant were removed and added to opaque bottom wells and tested for LDH activity using the commercial kit CytoTox-One homogeneous membrane integrity assay (Promega) according to the manufacturer's instructions.

##### Statistical Analyses

Statistical analyses (one-way ANOVA and Student's *t* test) were performed using the software GraphPad Prism 5.0, as described in the legend for each experiment.

## Results

### 

#### 

##### Characterization of AF- and PMA-induced NET Formation

**FIGURE 1.**
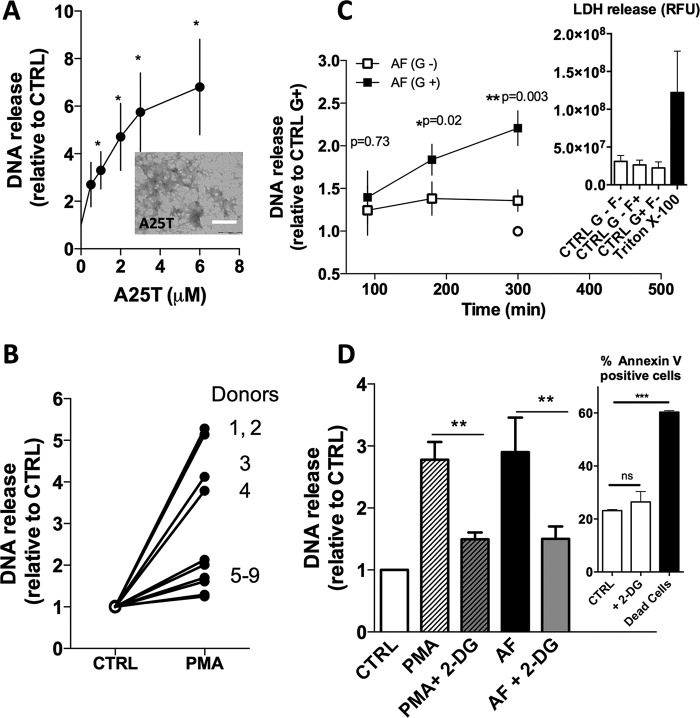
**Glucose is important for NET release by human neutrophils.** *A* and *B*, human neutrophils were stimulated with different concentrations of amyloid fibrils composed of transthyretin mutant A25T (A25T; *A* and *inset*), and NET release was assessed after 90 min. Neutrophils were also incubated with 100 nm PMA for 90 min, and NET release was assessed for each donor (*B*). *C*, neutrophils were incubated in HBSS containing no glucose or other carbon sources (*white symbols*; G^−^) or 5.5 mm d-glucose (*black symbols*; G^+^) and then stimulated with 3 μm AF for 1.5, 3, or 5 h (*squares*). Untreated neutrophils (CTRL) were incubated for 5 h (*circles*) in HBSS containing no glucose (*white circles*) or 5.5 mm glucose (*black circles*), and the supernatant containing the NETs was measured for extracellular DNA and compared with untreated neutrophils in HBSS containing glucose (CTRL G^+^; *black circles*). The *inset* in *panel C* shows viability of neutrophils monitored using LDH assay in untreated (CTRL) cells incubated for 5 h without glucose (CTRL G^−^F^−^) with 5.6 mm fructose (CTRL G^−^F^+^) or with 5.5 mm glucose (CTRL G+F^−^) or in cells that were lysed with Triton X-100 to determine the maximum LDH release for 100% cell death. *RFU*, relative fluorescence units. *D*, 2-DG inhibits DNA release induced by PMA or AF. Neutrophils were incubated with 2 mm 2-DG in RPMI 1640 and treated with 3 μm AF or 100 nm PMA for 3 h. The *inset* in *panel D* shows flow cytometry analysis of cells treated with 2-DG and annexin V. Data are means ± S.E. (*error bars*), total donor, *n* > 6. Statistical tests used were Student's *t* test or one-way ANOVA and Bonferroni's multiple comparison test where *, *p* < 0.05; **, *p* < 0.01 and ***, *p* < 0.001. *ns*, not significant. *Scale bar* in *panel A inset*, 5 μm.

As a NET inducer, initially we chose AF composed of A25T, a highly amyloidogenic variant of transthyretin that forms amyloid fibrils as observed by transmission electron microscopy (Fig. 1*A*, *inset*) and thioflavin-T binding (not shown (5)). As seen in Fig. 1*A*, the extent of DNA release as a measure of NET formation was dependent on the concentration of AF, beginning to level off at ∼3–6 μm AF. We also noticed that DNA release was time-dependent (5), reaching a maximum after 3 h of incubation in the presence of 3 μm AF, a time window in which the neutrophils remain viable in solution. We emphasize that the NET formation observed herein is donor-dependent for both AF-induced NET (5) and PMA-induced NET as seen in Fig. 1*B*, where 100 nm PMA was used as a NET inducer for 1.5 h. Although for some neutrophils the addition of PMA led to a very modest increase in DNA release (Fig. 1*B*, donors 5–9), in samples from other donors, there was a 5–6-fold increment (Fig. 1*B*, donors 1–4). It is unclear why neutrophils from some donors are more effective in producing NET. Based on these data, in the next experiments, we used 3 μm AF composed of A25T or 100 nm PMA during 1.5 h for NET induction, unless otherwise stated.

##### AF- and PMA-induced NET Formation Is Glucose-dependent

Neutrophils rely mainly on glycolysis to obtain the energy necessary for metabolic reactions and survival (22–24). Therefore, we asked whether NET could be induced effectively when neutrophils were deprived of glucose.

For this purpose, we incubated isolated human neutrophils in HBSS, a saline solution that contains Ca^2+^ and Mg^2+^, but no glucose or other source of carbohydrate, lipid, or protein (NCS). We compared the amount of NET released by these neutrophils with that released by neutrophils that were from the same donors but maintained in HBSS containing 5.5 mm d-glucose, a concentration similar to the RPMI 1640 medium used in previous experiments (5). Initially, both neutrophil populations were challenged with AF and DNA release was evaluated for a maximum of 5 h (Fig. 1*C*). After 1.5 h in the presence of AF, neutrophils incubated in the absence (G^−^, *white squares*) or in the presence of glucose (G^+^, *black squares*) showed similar amounts of DNA release, but after 3 or 5 h of induction, the neutrophils incubated in the presence of glucose continued releasing DNA, whereas the starved neutrophils did not present this enhancement (Fig. 1*C*). Viability assays (LDH) performed with untreated neutrophils (CTRL) incubated for 5 h in solutions containing no glucose or fructose (G^−^F^−^), only fructose (G^−^F^+^), or only glucose (G^+^F^−^) showed equal viability, suggesting that starvation or the absence of glucose do not compromise neutrophil viability (Fig. 1*C*, *inset*). Also, we tested whether an inhibitor of glycolysis, 2-DG, was able to inhibit NET release when the cells were incubated in the presence of glucose. As seen in Fig. 1*D*, AF- and PMA-treated neutrophils that were pretreated with 2-DG were not able to produce NETs, but were still viable (Fig. 1*D* and *inset*). Taken together these data suggest that NET formation might be a glycolysis-dependent process.

**FIGURE 2.**
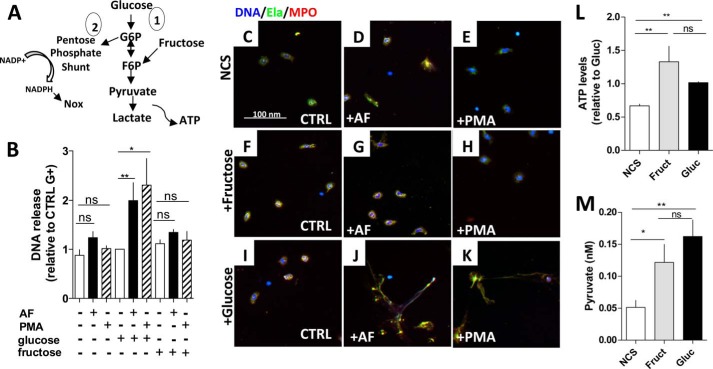
**Glucose addition, but not fructose, is able to sustain NET release in human neutrophils.** *A*, schematic drawing showing the simplified metabolic pathways that glucose or fructose might take inside human neutrophils. *B*, human neutrophils were incubated in HBSS containing no glucose, 5.5 mm d-glucose, or 5.6 mm d-fructose. Afterward, neutrophils were stimulated with 3 μm AF (*black bars*) or 100 nm PMA (*hatched bars*) for 3 h. Untreated neutrophils (*white bars*) were incubated for 3 h, and then all samples were measured for extracellular DNA and compared with untreated neutrophils in HBSS containing glucose (*CTRL G*^+^). *C–K*, neutrophils treated as shown in *panel B* were incubated with anti-human elastase (*Ela*; *green*), anti-human MPO (*red*), and a DNA marker (Hoechst; *blue*). Note that *NCS* means HBSS with no carbon source. *L* and *M*, untreated neutrophils were incubated in HBSS containing no glucose or other carbon source (NCS), 5.5 mm d-glucose (*Gluc*), or 5.6 mm d-fructose (*Fruc*), and lysates were tested for ATP (*L*) and pyruvate (*M*). ATP levels were normalized to neutrophils incubated in glucose. Data are means ± S.E. (*error bars*), total donor, *n* = 4–8. Statistical tests used were one-way ANOVA and Bonferroni's multiple comparison test where *, *p* < 0.05 and **, *p* < 0.01. *ns*, not significant.

When glucose enters the cell, it is readily phosphorylated by hexokinase, generating glucose 6-phosphate (G6P), which can flow through two different pathways: the glycolytic pathway, generating fructose 6-phosphate that is metabolized to lactate, producing ATP (Fig. 2*A*), or the PPP-forming ribulose-5-phosphate with the concomitant formation of NADPH (Fig. 2*A*, *routes 1* and *2*, respectively). It is important to recall that NADPH is important for Nox activity and ROS production (2), an element necessary in NET formation. In addition to the aforementioned fates of glucose, this sugar can also be used in glycogen synthesis and protein glycosylation (25, 26), but these fates of glucose were not addressed in the present study.

To assess the relative importance of glycolytic pathway and PPP for NET production, we performed a NET release experiment in which human neutrophils were stimulated with AF or PMA for 3 h in HBSS containing NCS, 5.5 mm glucose, or 5.6 mm fructose (Fig. 2*B*). Although it is not known whether neutrophils possess fructose transporters, we observed that fructose was able to enter neutrophils and generate ATP and pyruvate (Fig. 2, *L* and *M*, respectively) in levels comparable with those obtained in the presence of glucose. Also, previous data have shown that the uptake of [^14^C]fructose by neutrophils induces ^14^CO_2_ production, an indication that fructose is, to some extent, metabolized by neutrophils (27). Inside the cell, fructose is phosphorylated by hexokinase to form fructose 6-phosphate, which follows the glycolytic pathway and is metabolized into lactate and ATP (Fig. 2*A*) (28). Interestingly, because fructose 6-phosphate is downstream from the entry into PPP, when cells are incubated in its presence, we expect this pathway to be shut down, decreasing the levels of NADPH, an important element for Nox activity and NET formation.

As seen in Fig. 2*B*, significant DNA release was observed only when neutrophils were incubated in the presence of glucose and challenged by AF and PMA. Cells incubated in fructose or in HBSS containing NCS released NET similarly to unchallenged neutrophils (Fig. 2*B*). To confirm this observation, NET formation was evaluated through the use of a DNA marker (Hoechst; *blue*) and antibodies against neutrophil elastase (*green*) and MPO (*red*), two enzymes that decorate the NETs (Fig. 2, *C–K*). Neutrophils that do not form NETs remained circular and contained lobulated nuclei, and enzyme immunoreactivity was contained to the cytoplasm (Fig. 2, *C–H*). Again, only the neutrophils incubated with glucose and challenged with AF or PMA underwent NET formation (Fig. 2, *J* and *K*), whereas the cells incubated with NCS (Fig. 2, *D* and *E*) or fructose (Fig. 2, *G* and *H*) did not. Note that neutrophils treated with fructose or glucose produced similar amounts of ATP (Fig. 2*L*), but did not release NETs (Fig. 2, *B* and *F–H*), which suggests that an imbalance in the amount of ATP might not be the factor responsible for the ineffectiveness of fructose in inducing NET. These data indicate that glucose flow into the PPP, and not only the glycolytic pathway, is necessary for NET formation.

##### Evaluating ROS Production in AF- or PMA-stimulated Neutrophils in the Presence or Absence of Carbon Sources

**FIGURE 3.**
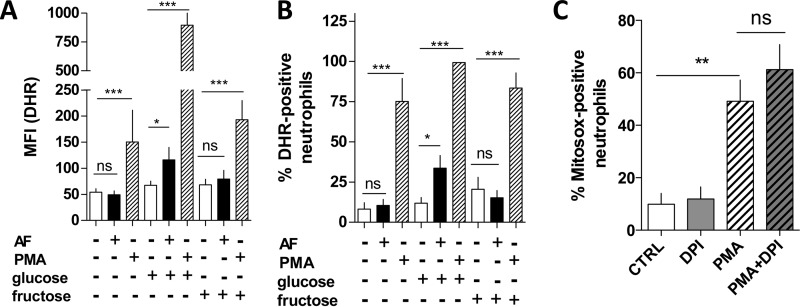
**Absence of glucose or addition of fructose decreased ROS production in human neutrophils stimulated with AF or PMA.** *A* and *B*, human neutrophils were incubated in HBSS containing no glucose, 5.5 mm d-glucose, or 5.6 mm d-fructose for 3 h. Afterward, neutrophils were incubated with DHR and then stimulated with 3 μm AF (*black bars*) or 100 nm PMA (*hatched bars*). Untreated neutrophils (*white bars*) were used as control, and then all samples were measured using flow cytometry for intracellular ROS. Here we show the mean fluorescence intensity of DHR (*A*) and percentage of DHR-positive neutrophils (*B*). *C*, neutrophils were incubated with MitoSOX, and afterward, PMA was added to the medium. Mitochondrial ROS was measured by quantifying the percentage of MitoSOX-positive neutrophils using flow cytometry. Data are means ± S.E. (*error bars*), total donor, *n* = 8–13. Statistical test used in *panels A* and *B* was one-way ANOVA and Bonferroni's multiple comparison test, and in *panel C*, Bartlett's post hoc test was used where *, *p* < 0.05; **, *p* < 0.01 and ***, *p* < 0.001. *ns*, not significant.

NET release induced by a variety of stimulants was shown to be dependent on ROS production by Nox (2, 4, 11), a cytoplasmic membrane-bound enzyme. To evaluate whether the PPP is indeed a source of NADPH for the generation of ROS by Nox and consequently to induce NET, we measured ROS production by flow cytometry (Fig. 3, *A* and *B*) using DHR, a fluorescent probe that can detect intracellular ROS/RNS.

Interestingly, the extent of ROS production was dependent on the stimulus used (AF or PMA) as well as on fuel source (glucose or fructose). As seen in Fig. 3*A* (*black bars*), AF was effective in inducing ROS production only when neutrophils were incubated in the presence of glucose, being ineffective either when cells lacked sugar or when only fructose was present. On the other hand, observing the MFI of DHR (Fig. 3*A*, *hatched bars*) or the percentage of the population of DHR-positive neutrophils as compared with untreated cells (Fig. 3*B*, *hatched bars*), we can affirm that PMA induced ROS production in all conditions, namely, in sugar-free solution as well as in the presence of glucose and fructose. However, it is important to note that the MFI from PMA-treated neutrophils incubated in the presence of glucose is 5-fold greater than PMA-treated cells in sugar-free solution as well as in the presence of fructose (Fig. 3*A*, compare *hatched bars*). These results suggest that glucose is needed to increase ROS production by neutrophils, possibly through the generation of intermediates, such as NADPH, to fuel ROS-producing enzymes like Nox.

##### Mitochondrial ROS Are Not Sufficient to Induce NETs

Because the respiratory complexes in mitochondria are important sources of ROS production in cells, we investigated whether PMA-induced ROS production observed in Fig. 3 might come from this organelle. To pursue this, we incubated PMA-treated human neutrophils in RPMI 1640 with a mitochondria-specific ROS detecting fluorescent probe (MitoSOX) in the absence and in the presence of DPI, a Nox inhibitor that can also inhibit other flavoproteins, such as complex I and II, but not complex III from the mitochondria (29). Interestingly, even in the presence of DPI, PMA-induced ROS production took place (Fig. 3*C*, compare *white hatched bars* with *gray hatched bars*), corroborating the idea that PMA might induce not only ROS from Nox, but also possibly from other sources such as complex III from mitochondria.

**FIGURE 4.**
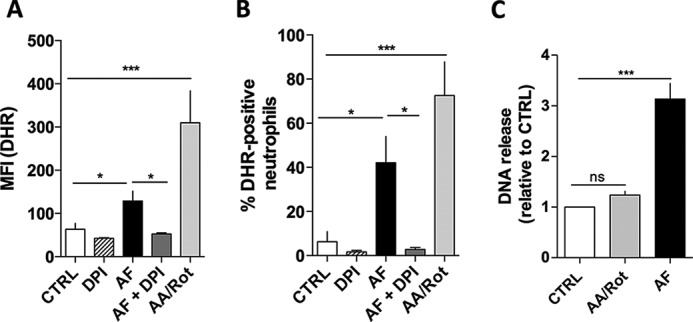
**Mitochondrial ROS is not effective in inducing NETs.** *A* and *B*, human neutrophils were incubated for 30 min in RPMI 1640 with complex I and III inhibitor Rot and AA (Rot/AA, *light gray bars*) or DPI (*hatched bars*) and then incubated with DHR. Neutrophils were also incubated with 3 μm AF (*black bars*) or 3 μm AF and DPI (*AF*+*DPI*, *dark gray bars*). Untreated neutrophils (*white bars*) were used as control, and then all samples were measured using flow cytometry for intracellular ROS. Here we show the mean fluorescence intensity of DHR (*A*) and percentage of DHR-positive neutrophils (*B*). *C*, we also evaluated NET release in samples pretreated with Rot/AA for 5 h alone (*light gray bars*), in samples pretreated with 3 μm AF for 5 h (*black bars*), or in untreated cells (CTRL, *white bars*). Results were plotted in comparison with untreated neutrophils (CTRL), and AF-treated neutrophils were used as positive controls. Data are means ± S.E. (*error bars*), total donor, *n* = 4–5. Statistical tests used were one-way ANOVA and Bonferroni's multiple comparison test where *, *p* < 0.05 and ***, *p* < 0.001. *ns*, not significant.

Next, we tested whether mitochondrial ROS were effective in inducing NET formation by incubating neutrophils in the presence of glucose with Rot and AA, inhibitors of complex I and III, respectively, and challenging the cells with AF. As expected, the addition of Rot and AA to neutrophils induced mitochondrial ROS production in considerable amounts (Fig. 4, *A* and *B*, *light gray bars*) (30, 31). Interestingly, AA/Rot addition, although inducing ROS release, was unable to generate a significant increase in NET formation, as measured by DNA release (Fig. 4*C*, *light gray bar*), which suggests that mitochondrial ROS alone do not trigger NET release.

As previously shown in Fig. 3, AF induced ROS production only when neutrophils were incubated in glucose-rich medium (for better comparison, these data were reproduced in Fig. 4, *A* and *B*, *black bars*), as well as DNA release (Fig. 4*C*, *black bar*), suggesting that ROS production and NET release, in this case, are NADPH-dependent. To corroborate these data, as controls, we demonstrated that AF-induced ROS production in neutrophils can be completely inhibited by DPI (Fig. 4, *A* and *B*, *dark gray bars*), as well as NET release, as shown before in our previous publication (5). Altogether, we strengthen the idea that NET formation might require a specific ROS species, which is generated by Nox, a NADPH-dependent enzyme, and not from the mitochondria.

##### G6PD, the First Enzyme of the PPP, Is Necessary for ROS Production and NET Release

**FIGURE 5.**
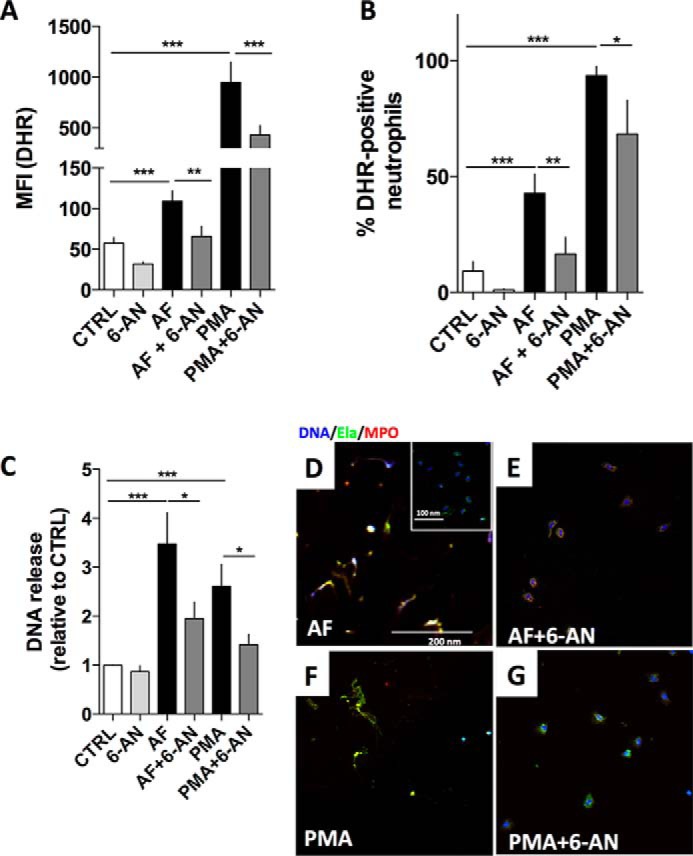
**The pentose phosphate pathway enzyme, G6PD, is necessary for NET formation.** *A* and *B*, human neutrophils were incubated for 30 min in RPMI 1640 with G6PD competitive inhibitor 6-AN and then incubated with DHR and 3 μm amyloid fibrils (AF) or 100 nm PMA. Untreated neutrophils (CTRL; *white bars*) were used as control, and then all samples were measured using flow cytometry for intracellular ROS. Here we show the mean fluorescence intensity of DHR (*A*) and percentage of DHR-positive neutrophils (*B*). *C*, we also evaluated NET release in samples pretreated with 6-AN for 30 min alone, followed by 3 μm AF or 100 nm PMA. Results are plotted in comparison with untreated neutrophils (CTRL). *D–G*, neutrophils treated as shown in *panel C* were incubated with anti-human elastase (*Ela*; *green*), anti-human MPO (*red*), and a DNA marker (Hoechst; *blue*). Data are means ± S.E. (*error bars*), total donor, *n* = 10–13. Statistical tests used were one-way ANOVA and Bonferroni's multiple comparison test where *, *p* < 0.05; **,*p* < 0.01 and ***, *p* < 0.001. *ns*, not significant.

The path that G6P takes through the PPP generates NADPH from the enzymatic reaction of G6PD, which uses G6P as substrate to produce 6-phosphogluconolactone, reducing NADP^+^ to NADPH. We hypothesized that NADPH from the G6PD reaction is needed to fuel Nox activity and produce ROS that are effective in signaling to human neutrophils to release NET under certain stimulus conditions. To test this hypothesis, human neutrophils incubated in the presence of glucose (RPMI 1640 medium) were pretreated for 30 min with the competitive inhibitor of G6PD, 6-AN (Fig. 5), and then stimulated with either AF or PMA to induce ROS production (Fig. 5, *A* and *B*). This inhibitor was not toxic to neutrophils (data not shown), but was able to reduce ROS production in both AF-treated and PMA-treated cells (Fig. 5, *A* and *B*, compare *black bars* with *dark gray bars*), although less effectively in PMA, possibly because PMA also induces the production of mitochondrial ROS that do not depend on NADPH from the PPP (Fig. 3, fructose condition). As expected, this diminished production of ROS in the presence of 6-AN was followed by a decrease in DNA release (Fig. 5*C*, compare *black bars* with *dark gray bars*) and NET formation evaluated by immunofluorescence using NET markers (elastase, *green*; myeloperoxidase, *red*; and DNA marker/Hoechst, *blue*; Fig. 5, *D–G*). Untreated cells (Fig. 5*D*, *inset*) or cells pretreated with 6-AN and then stimulated with AF (Fig. 5*E*) or PMA (Fig. 5*G*) did not produce visible NETs, whereas cells only treated with AF (Fig. 5*D*) or PMA (Fig. 5*F*) generated NETs, as expected. These data suggest that G6PD activity, and thus NADPH generation via PPP, is important for ROS production, and consequently NET release by human neutrophils.

##### Characterizing Neutrophil G6PD Activity in the Presence of AF and PMA

**FIGURE 6.**
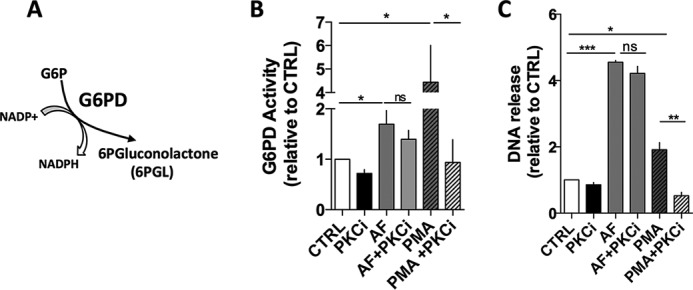
**G6PD activity is increased in AF- and PMA-treated neutrophils.** *A*, schematic drawing showing the enzymatic reaction catalyzed by G6PD. *B* and *C*, human neutrophils were incubated for 3 h in RPMI 1640 and then incubated with 3 μm AF (*gray bars*) or pretreated with PKCi for 30 min and then incubated with 100 nm PMA (*hatched bars*). Untreated neutrophils (CTRL; *white bars*) were used as control. G6PD activity was measured in 30 min measuring the optical density at 340 nm, and activity was calculated and normalized by CTRL samples (*B*). *C*, NET release was evaluated in samples shown in *panel B*, and results were plotted in comparison with untreated neutrophils (CTRL). Data are means ± S.E. (*error bars*), total donor, *n* = 10. Statistical tests used were one-way ANOVA and Bonferroni's multiple comparison test where *, *p* < 0.05 and **, *p* < 0.01. *ns*, not significant.

6-AN-induced inhibition of NET release suggests that a robust source of NADPH produced in the first step of PPP might be needed for effective ROS production by Nox (Fig. 2*A*), and consequently for NET production. Next, we asked whether AF and PMA would modulate G6PD activity in neutrophils. To address this question, neutrophils were activated with AF or PMA for 3 h and lysed at −80 °C, and the cell extracts were used to measure G6PD activity by following NADPH production at 340 nm at 30 °C by adding NADP^+^ and G6P (Fig. 6, *A* and *B*). Notably, G6PD activity increased ∼2- and 5-fold in neutrophils treated with AF or PMA, respectively, in comparison with untreated cells (CTRL, Fig. 6*B*). Because PMA is a PKC agonist (32), we pretreated neutrophils for 30 min with a PKC inhibitor (PKCi = chelerythrine), stimulated with PMA for 3 h, and then monitored both G6PD activity (Fig. 6*B*) and DNA release (Fig. 6*C*). PKCi altered significantly both G6PD activity and DNA release induced by PMA (Fig. 6, *A* and *B*, *hatched bars*). Interestingly, although PKCi was able to prevent PMA-induced increase in G6PD activity (Fig. 6*B*, compare *hatched bars*) as well as the production of NET (Fig. 6*C*, compare *hatched bars*), PKCi did not interfere with the AF-induced increase in G6PD activity (Fig. 6*B*, compare *gray bars*) or the associated NET production (Fig. 6*C*, compare *gray bars*). These data suggest that G6PD activity can be modulated by both AF and PMA, and only in the case of PMA, this modulation depends on PKC. Interestingly, we did not observe a direct correlation between G6PD activity and the amount of NET release (data not shown). However, G6PD activity is always detectable in neutrophils that release NETs.

**FIGURE 7.**
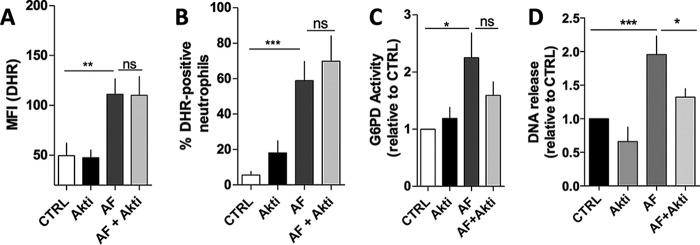
**Akt is necessary for AF-induced NET formation but not for ROS production or G6PD increased activity.** *A* and *B*, neutrophils pretreated with 10 μm Akti for 30 min and incubated with 3 μm AF were measured for intracellular ROS using flow cytometry and DHR probe. Here we show the mean fluorescence intensity of DHR (*A*) and percentage of DHR-positive neutrophils (*B*). *C*, G6PD activity was measured in samples shown in *panels A* and *B*, and activity was calculated and normalized by CTRL samples. *D*, NET release was measured in untreated cells (CTRL), cells pretreated with 3 μm AF alone (*AF*), or cells pretreated with Akti (*AF*+*Akti*) or Akti alone (*Akti*). Data are means ± S.E. (*error bars*), total donor, *n* = 6–8. Statistical tests used were one-way ANOVA and Bonferroni's multiple comparison test where *, *p* < 0.05; **, *p* < 0.01 and ***, *p* < 0.001. *ns*, not significant.

Recently, Akt kinase was implicated in NET formation as an essential signaling molecule that shifts apoptosis to NETosis in neutrophils stimulated with PMA (33). Akt is central to many signaling events and is a known survival signal when activated (33). Here we checked whether Akt signaling pathway participates in the AF-inducing NET release by using an Akti. Interestingly, as seen in Fig. 7, ROS production induced by AF was not modulated by Akti (Fig. 7, *A* and *B*). *Panel C* shows that the 2-fold enhancement of G6PD activity observed in neutrophils treated with AF was not completely prevented by Akti. Although some effect was observed when Akti-pretreated neutrophils were incubated with AF, this experiment did not reach statistical significance. These data are interesting because, as shown in Fig. 6, AF exerts a direct effect on PPP, especially by enhancing G6PD activity and NADPH production to fuel Nox to produce ROS to trigger NET release. Although Akt does not modulate ROS or G6PD activity, Akt participates in NET formation by AF-treated neutrophils (Fig. 7*D*), suggesting that AF-induced Akt activation might be downstream of ROS production and G6PD modulation by AF.

These data are in accordance with the data described by Douda *et al.* (33), where PMA-induced ROS production was also not modulated by Akti. These authors also showed that Nox-generated ROS influenced Akt phosphorylation in human neutrophils stimulated by PMA (33), suggesting that activation of Akt was also downstream to ROS production in this model.

## Discussion

NET is an important mechanism used by neutrophils to kill and trap microorganisms. It can also influence the innate and adaptive immune response (2) and is a key element in autoimmune response in diseases such as systemic lupus erythematosus (4, 11). Although many groups have described events related to NET release (2), the complex intracellular signaling involved in NET formation remains unclear for many NET inducers. The main theory for NET release suggests that Nox-derived ROS is essential to NET formation, and our study incorporates novel knowledge into this theory by showing that PPP and G6PD play an important role in NET formation: the PPP provides NADPH for maintaining Nox activity and ROS production. Nox-derived ROS, but not NADPH-independent ROS production by the mitochondria, are efficient in inducing NETs.

Neutrophils depend mainly on glycolysis for energy supply (22–24) due to the fact that these cells need to function in environments that sometimes lack oxygen (34). Also, neutrophil mitochondria are scarce and lack the respiratory chain supercomplex organization (22). Although neutrophil mitochondria produce significant amounts of ROS when stimulated by PMA (12, 35), our results are in agreement with those of Kirchner *et al.* (12), who demonstrated that two mitochondrial uncoupling agents and rotenone do not influence NET release by neutrophils stimulated by PMA. We believe that production of mitochondrial ROS, observed in our study, does not depend on glucose or NADPH derived from PPP because in the absence of glucose or after PPP blockage through incubation of neutrophils in a solution containing fructose or the G6PD inhibitor 6-AN, ROS release is not affected by cells stimulated with PMA. In the absence of any energy source, ROS production can be fueled by autophagy, which provides intermediates for the respiratory chain enzymes (36). Interestingly, we observed that human neutrophils stimulated with PMA and incubated in the presence of 6-AN, in the absence of glucose or in solution with only fructose added as an energy source, did not release NETs (Fig. 5). This suggests that although PMA induces the production of mitochondrial ROS that is not involved in NET signaling, it also needs the PPP to produce Nox-derived ROS, which is vital for NET release.

Interestingly, data from Saraiva and co-workers suggested that mitochondrial ROS is not effective in inducing NETs in PMA-treated neutrophils, as shown by using MitoTEMPO, a mitochondrial ROS scavenger. Under this condition, NETs are still produced.^6^ Moreover, neutrophils from patients with chronic granulomatosis disease, which do not have functional Nox, are able to produce mitochondrial ROS when stimulated with antimycin A, but their neutrophils do not produce NETs (35), also strengthening our observation on the absence of association between mitochondrial ROS and NET formation. The observation that treatment with AA/Rot induces DHR-dependent fluorescence (presumably of mitochondrial origin) but not DNA release (Fig. 4) raises questions about the mechanisms mediating ROS-induced NETs. Thus, because H_2_O_2_ diffuses in biological systems (and in these experiments it does because it is detected by DHR), in theory, even mitochondrial ROS should be able to induce NETs. Why do ROS from different sources seem to have different capacities to induce NETs? This is an important question that should be addressed in the future.

Unfortunately, it is not yet possible to establish a direct dose-response correlation between the amounts of ROS produced in response to a specific stimulus and the extent of NET formation. Also, the lack of tools to improve the detection of compartmentalized and highly reactive, short-lived ROS species prevented us from establishing a correlation between glucose availability, ROS production, and NET generation.

During the completion of this study, another study was published showing that PMA-induced NET formation, but not ROS production, depends on glucose (37), which is fully consistent with our findings. Moreover, in our study, we expand this observation to AF-induced NET formation, also evaluating the contribution of the PPP to NET release, which has not been explored so far. Interestingly, in the case of AF, our data showed that NET release and ROS production depend on glucose, suggesting a direct effect on Nox activity. This also suggests that different NET stimuli can elicit distinct signaling and metabolic pathways in neutrophils.

It has been shown by Douda *et al.* (33) that Akt might be a target for ROS signaling because Akt phosphorylation depends on Nox-derived ROS. Herein, we also observed that Akt signaling is important for NET release by AF and that Akt inhibition does not affect ROS production, suggesting that these signaling mechanisms might be independent of each other or that ROS production precedes Akt activation (33).

Clearly, as we observed, AF and PMA induce NETs by different mechanisms that might share common pathways: both AF and PMA need glucose, PPP, G6PD-derived NADPH, and Nox-derived ROS to efficiently form NETs. Although this common mechanism might also be shared by other NET inducers, the complexity of NET intracellular signaling is impressive and very intricate. Here we also observed that NET formation might not need ATP because neutrophils given fructose as a source of energy do not release NETs, but produce ATP similarly to neutrophils incubated in a glucose-only medium. However, we cannot discard that ATP is being used in NET signaling because the fluctuations of ATP levels may only be detected by more sensitive methods.

As mentioned before, in this study, we also observed that G6PD, the first enzyme in the PPP, is important for NET release as inhibition of G6PD by 6-AN decreases NET formation by neutrophils. In addition, the activity of G6PD is increased by AF- and PMA-treated neutrophils (Fig. 6), which could be by a direct or indirect mechanism. We observed that in the case of PMA, the increase in G6PD activity is inhibited when a PKC inhibitor is added to human neutrophils. G6PD has several phosphorylation sites, and some of them were shown to be phosphorylated by PKC (38), suggesting that this might be one mechanism by which PKC modulates G6PD activity in our model. In the case of AF, we observed that NET formation depends on Akt signaling. However Akt does not modulate G6PD activity significantly as PKC does in the case of PMA, suggesting that these mechanisms might not be linked to each other. Additionally, it is unclear whether ROS might modulate G6PD activity, but more studies are aimed to answer this question. PMA is a PKC agonist, which explains why most PMA-induced effects can be modulated by PKC inhibitors. However, in the case of AF, it remains unclear how neutrophils recognize AF. We believe that one or more receptors might be involved in AF recognition, but more studies need to be performed to address this question.

Understanding the participation of G6PD in NET release is clinically important because G6PD deficiency is the most common enzyme deficiency worldwide, affecting more than 400 million people (39). G6PD deficiency causes favism and acute hemolytic anemia and can be life-threatening (39). The activity of G6PD in these disorders depends mainly on the location of the mutation and can vary in patients from less than 1% of the normal values to 90%, which is considered asymptomatic (39). In 2013, it was shown that in neutrophils from Taiwan-Hakka type G6PD deficiency, ROS production is not disturbed (10) and NET formation occurs when the cells are stimulated with PMA (10). However, in these patients, the G6PD activity is close to 50% of the normal value (10), which might explain why G6PD activity does not influence NET release in this case. In our study, we completely inhibited G6PD activity using 6-AN or we decreased the amount of substrate by giving fructose to neutrophils, which may cause more severe effects than those described by Cheng *et al.* (10). Our data also open new questions such as how neutrophils and NETosis are regulated during conditions where oxidative stress is present, such as exercise, diabetes, aging, and even neurodegenerative diseases such as Alzheimer disease. The newly discovered connection between the brain and the peripheral lymphatic system (40) raises new questions about the role of peripheral leukocytes in neurodegenerative diseases.

In conclusion, we have shown that the metabolic shift to the PPP is important for NET release because G6PD, a major enzyme from PPP, has a relevant role in fueling Nox with NADPH to produce an effective species of ROS and thus induce NETs. We also showed that mitochondrial ROS release, which is NADPH-independent, is not effective in signaling to neutrophils to produce NETs. These data shed new light on understanding novel pathways involved in NET release and how neutrophil glycolytic metabolism contributes to NET formation.

## Author Contributions

E. P. A. designed the research, performed research, analyzed data and wrote the manuscript. A. B. G. C., N. C. R, T. S. S. V., and J. G. assisted with research, data analysis, and discussions. F. L. P., E. M. S., and D. F. analyzed data and wrote the manuscript.

## Footnotes

6 E. M. Saraiva *et al.*, submitted for publication.

## Footnotes

5 The abbreviations used are:

ROS: reactive oxygen species
RNS: reactive nitrogen species
G6P: glucose 6-phosphate
G6PD: glucose-6-phosphate dehydrogenase
6-AN: 6-aminonicotinamide
AF: amyloid fibrils
PMA: phorbol 12-myristate 13-acetate
PPP: pentose phosphate pathway
NET: neutrophil extracellular trap
MPO: myeloperoxidase
Nox: NADPH oxidase
LDH: lactate dehydrogenase
Rot: rotenone
AA: antimycin A
2-DG: 2-deoxyglucose
HBSS: Hanks' balanced salt solution
DPI: diphenyleneiodonium
DHR: dihydrorhodamine-123
PKCi: PKC inhibitor
Akti: Akt IV inhibitor
NCS: no carbon source
ANOVA: analysis of variance
MFI: mean fluorescence intensity
CTRL: control.
